# Design and Development of Hybrid Hydrogels for Biomedical Applications: Recent Trends in Anticancer Drug Delivery and Tissue Engineering

**DOI:** 10.3389/fbioe.2021.630943

**Published:** 2021-02-17

**Authors:** Mao-Hua Cai, Xiao-Yi Chen, Luo-Qin Fu, Wen-Lin Du, Xue Yang, Xiao-Zhou Mou, Pei-Yang Hu

**Affiliations:** ^1^Department of General Surgery, Chun'an First People's Hospital (Zhejiang Provincial People's Hospital Chun'an Branch), Hangzhou, China; ^2^Key Laboratory of Tumor Molecular Diagnosis and Individualized Medicine of Zhejiang Province, People's Hospital of Hangzhou Medical College, Zhejiang Provincial People's Hospital, Hangzhou, China; ^3^Clinical Research Institute, Zhejiang Provincial People's Hospital of Hangzhou Medical College, People's Hospital, Hangzhou, China; ^4^Department of Traumatology, Tiantai People's Hospital of Zhejiang Province (Tiantai Branch of Zhejiang People's Hospital), Taizhou, China

**Keywords:** hybrid hydrogels, design and development, biomedical applications, cancer drugs, tissue engineering

## Abstract

The applications of hydrogels in biomedical field has been since multiple decades. Discoveries in biology and chemistry render this platform endowed with much engineering potentials and growing continuously. Novel approaches in constructing these materials have led to the production of complex hybrid hydrogels systems that can incorporate both natural and synthetic polymers and other functional moieties for mediated cell response, tunable release kinetic profiles, thus they are used and research for diverse biomedical applications. Recent advancement in this field has established promising techniques for the development of biorelevant materials for construction of hybrid hydrogels with potential applications in the delivery of cancer therapeutics, drug discovery, and re-generative medicines. In this review, recent trends in advanced hybrid hydrogels systems incorporating nano/microstructures, their synthesis, and their potential applications in tissue engineering and anticancer drug delivery has been discussed. Examples of some new approaches including click reactions implementation, 3D printing, and photopatterning for the development of these materials has been briefly discussed. In addition, the application of biomolecules and motifs for desired outcomes, and tailoring of their transport and kinetic behavior for achieving desired outcomes in hybrid nanogels has also been reviewed.

## Introduction

**Figure 1:**
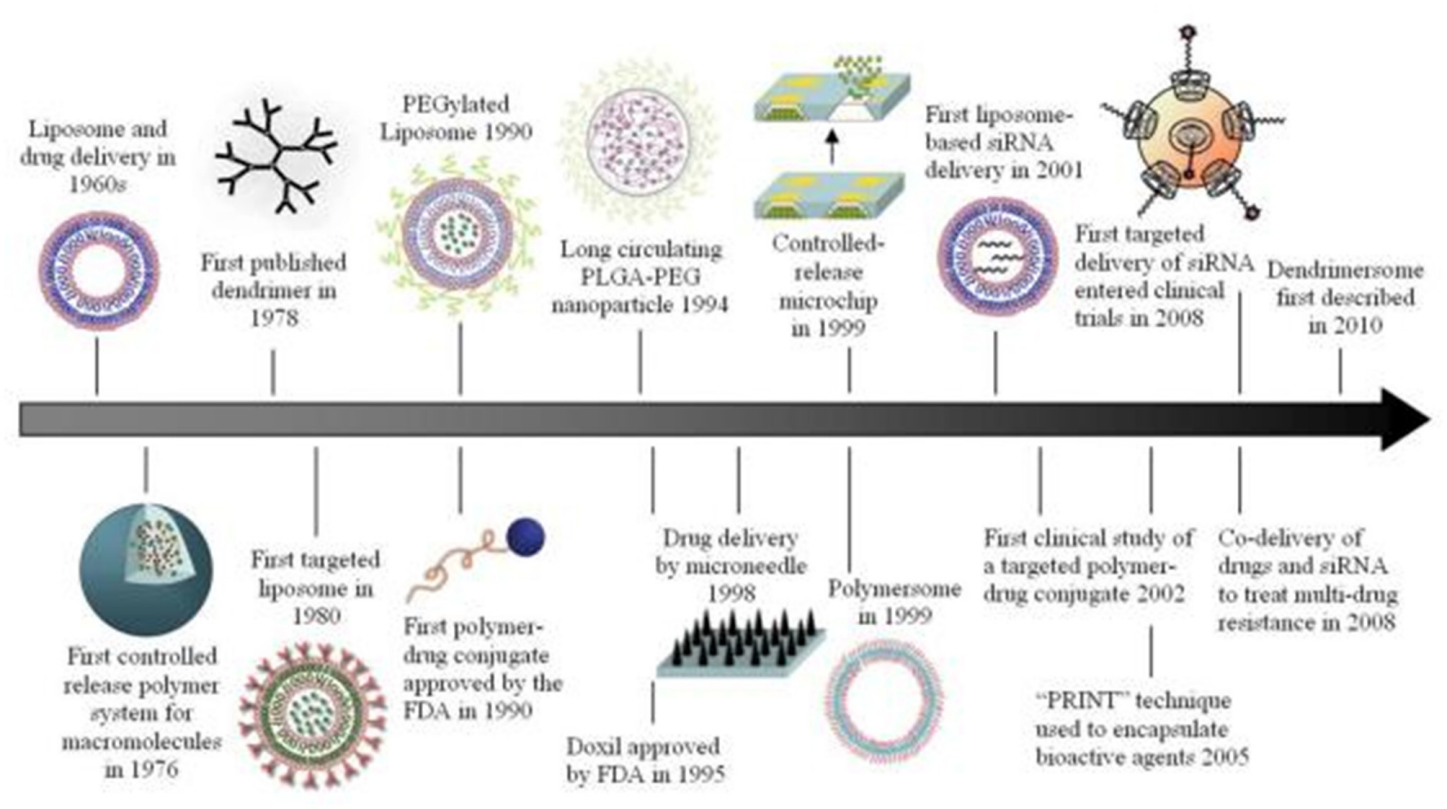
Different types of inorganic/organic nanomaterials and similar other devices that have been used as carrier systems for drugs or other bioactive substances. Reproduced with permission from Shi et al. (2010). Copyright (2010) American Chemical Society.

The use of nanotechnology in medicine, known as nanomedicine, offers several exciting possibilities in healthcare. The pharmacological properties such as circulating half-life and the solubility of drug molecules can be dramatically enhanced by employing nanoscale delivery vehicles (Shi et al., 2010). Nanotechnology has played a crucial role in the development of drug delivery systems since liposomes were first identified in the 1960s and adopted as protein and drug carriers for the treatment of various diseases (Bangham and Horne, 1964). As delivery vehicles, a range of inorganic/organic nanomaterials and other similar devices have been used to set up efficient therapeutic modalities (Figure 1). To date, the Food and Drug Administration (FDA) has licensed more than two dozen therapeutic products based on nanotechnology for clinical usage while some more are in clinical trial stages (Wagner et al., 2006; Zhang et al., 2008; Davis et al., 2010). Of these substances, most follow non-target delivery mechanisms (e.g., polymers and liposomes) and are thus known to be nanotherapeutics of the first generation (Riehemann et al., 2009).

Nanosystems of the first generation offers several benefits relative to traditional drug delivery. In general, they can improve therapeutic effectiveness by extending drug half-life, increasing hydrophobic drug solubility, decreasing potential immunogenicity, and/or releasing drugs in a prolonged or stimulated manner. Furthermore, because of the enhanced permeability and retention (EPR) effect in specific tissues such as tumors, nanoscale particles can tend to grow passively (Maeda, 2010). In addition to these clinically efficient nanosystems, nanotechnologies have been used to facilitate new therapies and to create “hybrid” and “smart” nanosystems for next-generation biomedical applications (Shi et al., 2010).

Hydrogels are networks of hydrophilic polymer that can retain, expand, and carry large volumes of liquid in the simplest form (Shi et al., 2010). Hydrogels are innately well-suited for biological applications with their elevated permeability, and water content as well as their structural and viscoelasticity resemblance to the cell membrane. As demonstrated by Wichterle and Lim in 1960, these main features make them appealing for biomedical use as well as continue to exist as hubs for tissue engineering and drug delivery (Bangham and Horne, 1964). Enhanced material versatility and function have been facilitated by chemical advances that enable the integration of molecules that can guide cellular activities. Moreover, it also enables the hydrogel system to be activated under time control, as well as allow the incorporation of transporters such as nanoparticles and microstructured domains into the hydrogel network.

Hydrogels typically comprise polar or functional groups with specific charges that give them hydrophilicity, water absorption ability, and, therefore, swell up in a particular medium with improved vulnerability to stimuli, etc. (Durmaz and Okay, 2000; Ekici and Saraydin, 2004). The concept of Hybrid Hydrogels remains debatable. They either are described as complicated, consisting of hundreds of physically or chemically cross-linked nanogels (Zhang et al., 2018), or they relate to structures made up of various nanoparticles and/or polymers, such as magnetic, carbonaceous, and plasmonic nanoparticles. It has also been defined as a system comprising of building blocks that are functionally, morphologically, and chemically different from at least two groups related by physical or chemical means. It might involve biologically active polymers, such as pro-polysaccharides and/or polysaccharides hybridization can occur at microscopic or molecular levels depending on the size and role of the basic components (Jia and Kiick, 2009; Molina et al., 2015).

Each medical approach provides for the specific selection of a combination of composite material to match the required functional and structural properties with a new profile that must be effectively generated by an advanced polymer device (Myung et al., 2008). The combination of protein and other polymers is one of the most important examples. Conjugation or polymerization (click chemistry) with synthetic polymers may lead to such combinations, resulting *in vitro* and *in vivo* compatible hybrid hydrogels for application in drug delivery studies, wound healing and tissue engineering applications (Jonker et al., 2012; Lau and Kiick, 2015), or the extraction of growth factors from the extracellular environment (Maisani et al., 2017). Hybrid hydrogels are typically heterogeneous and are important to ensure adequate cell organization, cell-cell interactions, and adhesion for medical purposes (Yang et al., 2016; Joseph et al., 2018; Patel et al., 2018).

Owing to their versatile properties, hybrid hydrogels have got increasing scientific interest for diverse applications in biomedical fields. Recent advancements in hybrid hydrogels have led to their potential applications in the delivery of cancer therapeutics, and re-generative medicines. In this review, recent trends in advanced hybrid hydrogels systems incorporating nano/microstructures, their synthesis, and their potential applications in tissue engineering and anticancer drug delivery are discussed. Examples of some new approaches including click reactions implementation, 3D printing, and photopatterning for the development of these materials, the application of biomolecules and motifs for desired outcomes are reviewed.

## Hybrid Nanogels

Subsequently formed hybrid nanogels are highly crosslinked nano-sized systems (Dorwal, 2012; Yadav et al., 2017) having a size <100 nm (Bencherif et al., 2009) and a non-fluid colloidal/polymer network which incorporates the characteristics of both nanomaterials and hydrogels. Their nanoscale offers a broad bioconjugation surface area, a prolonged circulation duration in the blood, and the potential to be actively or passively focused at the specified area of action such as tumor sites (Molina et al., 2015). Smart hybrid hydrogels/nanogels have the capability to efficiently respond via changes in their refractive index, volume, and hydrophobicity/hydrophilicity, etc. to biomedically relevant changes such as temperature, glucose, pH, electrical field, magnetic field, light, ionic force/concentration, redox environment, chemicals, or specific biomarkers, etc. Micro-and nano-sized hydrogels are quicker than their macroscopic or bulk alternatives in responding to environmental changes that can be used more effectively in biomedical and sensing applications (Sahiner et al., 2006).

### Multifunctional Hybrid Nanogels

In the field of medicine/nanomedicine, multifunctional hybrid nanogels have identified uses of consistent optical sensing of specified stimuli in complex samples such as bioreactor fluids, blood and intracellular imaging, leading to the clarification of complex biological processes, the development of innovative diagnostics, and clinical applications (Wu and Zhou, 2010).

### Hybrid Polymer Nanogel/Hydrogels

Hybrid polymer nanogel/hydrogels include interpenetrated networks (IPNs) and core-shell particles. The core-shell strategy is especially useful for targeting therapy, while the interpenetration allows the development of multi responsive nanogels and the control of the drug release profile (Lohani et al., 2014).

### Physical Hydrogels

Physical hydrogels result by ionic and physical interactions, such as hydrogen bonds, coordination bonds, electrostatic and hydrophobic interactions in certain conditions and physico-chemical interactions (stereo-complexation, charge condensation, or supramolecular chemistry) (Hoare and Kohane, 2008). By changing the temperature, pH, ionic strength, or solvent composition, they mostly form a homogeneous solution and re-gel when they return to their initial conditions, being reversible gels, generally unstable and mechanically weak (Ebara et al., 2014). Physical hydrogels are not all being reversible gels with physical stimuli, such as Chitosan/GP gel. The physical cross-links are also formed by crystallization (Amini and Nair, 2012), between amphiphilic block and graft copolymers (Jin, 2012), and protein interactions (Augst et al., 2006). Physically crosslinked hydrogels show stimuli-responsiveness and self-healing properties, but their mechanical strength is low and they often exhibit plastic flow (Czarnecki et al., 2016).

### Chemically or Covalently Crosslinked Hydrogels

Chemically or covalently crosslinked hydrogels with a permanently fixed shape at rest, exhibit a low fracture toughness and extensibility. Therefore, it is preferred to create both physically and covalently crosslinking hydrogels (Sun et al., 2012; Lin et al., 2015), resulting doubly-crosslinked hybrid gels that combine all mentioned properties (Narita et al., 2013). Many double network (DN) hydrogels prepared by double chemically crosslinking or by hybrid physical/chemical crosslinking imply crosslinking agents, but they present toxicity which is an important disadvantage (Vasile et al., 2020). Some derivatives of DN hydrogel, not hydrogel itself are toxic and the toxicity of such system is due to the use of crosslinking agents. Designing a new generation of DN gels comprising two non-covalent associated networks is a promising technique.

Kondo and coworkers (Kondo et al., 2015) prepared a dually-crosslinked polymer gel with a very homogeneous network architecture, using a tetra-arm star-shaped poly(ethylene glycol) (PEG), PEG and poly(dimethylsiloxane) (PDMS) building blocks linked by orthogonal cross-coupling, The obtained network from hydrophilic and hydrophobic components regularly and uniformly distributed is non-covalent hydrophobic association whose strength is tuned by the molar ratio of the hydrophilic PEG and the hydrophobic PDMS segments (Sletten and Bertozzi, 2009).

### Self-Assembling Hybrid Hydrogels

Self-assembling hybrid hydrogels containing peptides provide the desired biological functionality and biodegradability, are able to mimic biological structures and materials having direct biomedical applications, namely as carriers for drug and cell delivery (e.g., incorporation of bioactive sequences from natural proteins). To control mechanical, biocompatibility and degradation properties, the peptides are combined with polymeric networks (Kopeček and Yang, 2012; Rodriguez et al., 2016) by chemical modification, covalently linking or non-covalent interactions between peptides and polymers (Baker and Chen, 2012).

Hybrid hydrogels self-assembled from graft copolymers via formation of coiled coil antiparallel heterodimers was also demonstrated, based on HPMA copolymers backbone and a pair of oppositely charged peptide grafts. The formation of these hybrid hydrogels was reversible (Yang et al., 2006). ADNA/poly(lactic-co-glycolic acid) (PLGA) hybrid hydrogel (HDNA) was prepared for water-insoluble ophthalmic therapeutic delivery of dexamethasone and it may be applied in treatment of various eye diseases (Ren et al., 2019).

## Methods of Preparation of Polymeric Hybrid Hydrogels

### Routes for Hybrid Hydrogels

Crosslinking approaches may include: (i) physical crosslinking (employing frequent freezing/thawing cycles leading to cryogels) through the complex process of coacervation, H-bonding, or ion interaction; (ii) grafting or chemical crosslinking by co-polymerization, polymerization, and chemical conversion (via employing crosslinking agents such as glutaraldehyde, glyoxal, borates, etc., and (iii) grafting or crosslinking through irradiations (gamma radiation or electron beam, based on the dose of the irradiations). The characteristics of hydrogels can be determined by various parameters, such as the structure, the shape of the cross-link, the final density, and the development of polymers, whereas environmental factors (such as ion power, pH, temperature, etc.) can be regulated for physical hydrogels.

Chemically cross-linked gels are prepared by reverse microemulsion, irradiation (ultraviolet, high-energy radiation via employing electron beam, or gamma), emulsion, radical polymerization/crosslinking, inverse mini-emulsion, heating, photolithographic chemical reactions via cross-linker as di-sulfide cross-linking, ionic, click chemistry (Thiol-ene couplings, Azide-alkyne cyclo-addition reactions, Tetrazine-norbornene chemistry, and Diels-Alder reactions), Schiff base crosslinking with a huge ensemble of reactions including Nucleophile addition and Michaelis-Arbuzov reaction (Gulrez et al., 2011), Michael type reactions and enzymatic cross-linking (Khademhosseini and Langer, 2007). For hydrogel preparation, both physical and chemical cross-linking methods are used (Budama-Kilinc et al., 2018).

The use of living/controlled radical polymerization techniques like iodine-mediated polymerization (RITP) degenerative chain transfer polymerization, reversible polymerization of addition-fragmentation chain transfer and catalytic atom (group) transfer radical polymerization (ATRP) is a transition toward the development of complex structures with a significant degree of versatility and configurational range (Barner-Kowollik, 2008). A new technique of hybrid hydrogel synthesis is required for the non-covalent binding of genetically engineered coiled-coil protein motifs to the hydrophilic synthetic HPMA copolymer backbone. The coiled-coil domains' self-assembly provided the physical crosslinking (Wang et al., 2001).

#### Chemical Modifications

Chemical changes include a variety of ligands that can be used for the delivery of targeted drugs, stimulation of release of the drug, or the preparation of complex materials. Lim et al. have documented the cross-linking of the hybrid network and the conjugation of proteins to the gel backbone as a functional protein immobilization platform (Lim et al., 2019).

#### Functionalization

Surface functionalization of hybrid hydrogels/nanogels can also be accomplished with specific ligands for achieving targeted therapy and reducing the toxicity (Sierra-Martin and Fernandez-Barbero, 2015). This type of functionalization is also critical for the development of various types of micro/macro/nanogel morphologies, such as hallow, core-and-shell, multilayer microgels, hairy microgels (Sanson and Rieger, 2010), etc.

#### Stealth Functionalization

A non-secondary requirement is needed for hybrid nanosystems/nanogels for biomedical purposes and drug delivery, as their biocompatibility is required both to minimize the organism's immune or inflammatory responses and to increase the supply of blood, biodistribution, and bioavailability of the transported drugs. Hybrid nanogels must be specifically aimed and explicitly constructed to accomplish this requirement. A very large range of architectures emerges from their functionalization, modification, and decoration (Kabanov and Vinogradov, 2009). Similarly, their modification can also be achieved via their conjugation with both inorganic (Karg, 2016) and organic (Wu and Wang, 2016) forms of nanostructures and nanoparticles. Hybrid nanogel reveal diverse morphologies depending on both the assembly techniques and the particle form, each element either being core or shell, of varying architecture and size (Eslami et al., 2019). These different morphologies can be achieved by physical crosslinking or chemical reactions, depending on ionic interactions, hydrogen bonds as well as other intermolecular bonds. Appropriate biocompatibility and surface decoration are, therefore, important parameters that greatly influence the biodistribution, along with the components, the surface charge, and the interaction of the ligands. Numerous covert functionalization, such as polyethylene glycols or chitosan, manipulate hydrophilic polymeric chains have been used for stealth purpose.

#### PEGylation

The process of PEGylation is carried out for increasing lifespan of circulation of nanostructures, thus leading to improved bioavailability of the drugs loaded in these nanostructures (Grover et al., 2013). A protein corona is developed around the antifouling PEG functionalization following this modification (Foster, 2010). It would create a restricted zone around the nanoparticles and reduce the enclosure of plasma proteins and the subsequent absorption of macrophages. PEGylation depends on a variety of factors including the hydrophilic characteristics of the PEG chains as well as molecular weight in 2000-13,000 Da range.

## New Approaches for the Development of Hybrid Hydrogels

Current advances in contemporary polymer chemical transformations have made it possible to develop advanced material systems. In general, “click reactions” (Thiol-norbornene click reaction, and Thiol-maleimide Michael addition) are highly helpful as they are orthogonal to many natural chemical functions, have few by-products, and can be tuned to demonstrate reversible and dynamically correct relationships with the resulting thioether succinimide linkage (DeForest and Anseth, 2011; Koshy et al., 2016). They are especially useful for the development of hydrogels with tunable viscoelasticity, high biocompatibility, and most significantly, for their capability to transport, protect and sensitively release their loaded therapeutic contents in the surrounding tissue (triggered by changes in pH or by the action biological substrates) (Baldwin and Kiick, 2011). For instance, hyaluronic acid hydrogels based on copper(I)-catalyzed azide-alkene cycloaddition (CuAAC) have been employed as cell scaffolds as well as repositories for the drugs (Crescenzi et al., 2007). In addition, the toxic catalysts employed in copper-catalyzed reactions can be eliminated or reduced with copper-free click chemistries like those used in azide-alkyne cycloaddition (Jiang et al., 2015), in radical-mediated thiol-ene/yne chemistry (Zhu et al., 2015), the Diels-Alder reaction (Fan et al., 2015), tetrazole- alkene photo-click chemistry (Fan et al., 2013), and the oxime reaction (Mukherjee et al., 2015).

Although hydrogels have key properties that make them ideal for biomedical applications such as tissues engineering and drug delivery (Jia and Kiick, 2009; Slaughter et al., 2009), it is still important to optimize their mechanical power, degradation/ release kinetics, as well as the bioactivities of loaded therapeutic contents. There are major heterogeneity and network defects in typical synthetic hydrogels such as phase-separated regions, entanglements, and chain ends which have a dramatic effect on mechanical properties and modify the biological activity and diffusion rates of active molecules (Jia and Kiick, 2009). Hybrid hydrogels are, however, designed to solve the problems associated with the development of current formulations. Further, they also extend a variety of applications from medical (spatial-temporally regulated profiles of drug release) (Chivers and Smith, 2017; Wang et al., 2018) to optoelectronics and magnetic materials (high-tech applications such as the manufacture of high-charge organic electronic devices having excellent properties related charge transport) (Babu et al., 2014; Amabilino et al., 2017). Hybrid hydrogels are made up of building blocks that are chemically, functionally, and morphologically different and may include biologically active peptides, proteins, or micro/ nanostructures that are interconnected by chemical or physical means. In general, peptides and proteins that are integrated into networks react with synthetic polymers through conjugation or polymerization (click chemistry) approaches to generate *in vitro* (cells proliferation, differentiation and migration studies) and *in vivo* (tissue engineering, drug delivery, and wound healing) compatible hybrid hydrogels (Jonker et al., 2012). The encapsulation of micro/ nanostructures within the hydrogels can be accomplished through chemical or physical encapsulation to improve the yield of mechanical reinforcement and the controlled delivery of cargo (Lau and Kiick, 2015) or sequestration of growth factors from the surrounding setting (Maisani et al., 2017). In addition, the heterogeneity of hybrid hydrogels will enhance cells organization, adhesion, and interactions between cells to generate tissue constructs having enhanced electroactivity, mechanical integrity, and cell organization (Shin et al., 2013; Xavier et al., 2015).

## Release Mechanisms of Bioactive Molecules From Hybrid Hydrogels

In a hydrogel network, the inclusion of micron-sized domains or particles enables improved structural integrity and can eventually serves as a tool for guiding cell behavior. Cells are known to show enough sensitivity to material properties such as size, modulus, pore, and elasticity, so it is of great interest to use these cell-modulating material properties for developing materials that facilitate growth, singling, proliferation, migration, and expression of cells (Vikingsson et al., 2015).

They are usually isotropic materials, despite various excellent properties of hydrogels for drug release, which can only experience uniform contraction and volumetric expansion in response to stimuli (Jeon et al., 2017) in contrast to different human tissues. Hybrid hydrogels that contain domains of micron-sized have been designed for maintaining cellular development, promoting microstructure-mediated cell migration, and release of their loaded bioactive agents in a regulated way under the influence of naturally occurring microscale structural motifs.

Micron-sized particles, including nanoparticles, can be integrated into a hydrogel network covalently or non-covalently to generate enhanced mechanical strength, release bioactive molecules, and facilitate a range of anticipated cellular responses. In general, the release of the loaded therapeutic substances from hybrid hydrogels can happen via one or more of three mechanisms: *(1) The burst or abrupt release that occurs as result of dissolution of the drugs absorbed on the surface of hydrogel; (2) release of the drugs due to their diffusion via the micro-particle or hydrogel matrix;* and *(3) release of the drugs due the hydrogel degradation* (Mahmoudian and Ganji, 2017). How the hydrogel releases the drug is often essential to achieve desirable therapeutic outcomes, and the required duration of drug availability (short term vs. long term) and its release profile (continuous vs. pulsatile) depend on the specific application. When the drug is exhausted, the hydrogel should be designed to either degrade to avoid surgical removal, or to be re-used by drug refilling. The degradation of the hydrogel may also need to be tailored to coordinate with tissue regeneration. Besides the general requirements, there exist other application-based requirements. For example, in the treatment of skin wounds, hydrogels are placed on dynamic surfaces to which they need to be adhesive and conform, while being tough enough to tolerate the surface movement (for e.g., strain of knee bending up to 50%) and deformation derived from the environment (for e.g., compression and scratching) (Burczak et al., 1994; Kong et al., 2004; Mahmoudian and Ganji, 2017). Hydrogels consist of a cross-linked polymer network, and open spaces (that is, meshes) between polymer chains; the meshes allow for liquid and small solute diffusion. Typical mesh sizes reported for hydrogels range from 5-100 nm, (Garcia and Kiick, 2019) and a number of approaches exist to determine the mesh size. Mesh size also play a key role in controlling diffusion and release of the bioactives from hydrogel system (Burczak et al., 1994). Another strategy to control the release of drug molecules initially entrapped in a hydrogel is to regulate network degradation. The mesh size increases as the network degrades, allowing drugs to diffuse out of the hydrogel. Degradation can occur in the polymer backbone or at the cross-links, and is typically mediated by hydrolysis (Kong et al., 2004; Boontheekul et al., 2005; O'Shea et al., 2015) or enzyme activity. An alternate strategy to release entrapped drugs is the controlled swelling of hydrogels. As a hydrogel swells, the mesh size increases. The extent of swelling of a hydrogel is a balance between forces that constrain network deformation and the osmosis that leads to water absorption (Brannon-Peppas and Peppas, 1991; Hong W. et al., 2008). The swelling behavior can be sensitive to various external conditions, including temperature (Hirokawa and Tanaka, 1984), glucose (Kokufata et al., 1991; Obaidat and Park, 1997), pH (Zhang et al., 2015), ionic strength (Ohmine and Tanaka, 1982), light (Yan et al., 2012), and electric fields (Murdan, 2003). These cues have been widely exploited in drug delivery. A final approach to release entrapped drug molecules is to mechanically deform the network, as this can both increase the mesh size by changing the network structure and trigger convective flow within the network (Huebsch et al., 2014). This strategy can generate pulsatile release patterns with fine control over the magnitude of the instantaneous release rate (Huebsch et al., 2014) using purely mechanical deformation, or using ultrasound and magnetic field-induced deformations.

These bioactive molecule release mechanisms include handles that can be modulated to affect transport; particle composition and polymer network can therefore be controlled to regulate mesh size and chemical degradation rates, and bio-responsive domains can be integrated to allow cell-responsive actions (Rehmann et al., 2017; Lau et al., 2018).

For instance, when vancomycin hydrochloride HMPC nanoparticles were included in a hydrogen based on chitosan/glycerophosphate, there was a noticeable effect of prolonging the drug release due to the diffusion of the transport mechanism (Mahmoudian and Ganji, 2017). In this situation, the drug release is slowed by the need to be diffused into both the hydrogel network and the microparticles. In another study, the drug release from carbohydrate based injectable hydrogel containing anionically functionalized microgels [poly(NIPAM functionalized with acrylic acid)] was studied (Sivakumaran et al., 2011). The release of loaded bupivacaine was regulated in this system not only by diffusive contributions, such as the hydrogel crosslinking density but also by the drug's partitioning affinity between the microgel phases and the bulk, which permitted minimization of the initial rapid release. In a study, a series of hydrogels made of self-assembling peptides, five graphene derivatives (GDs), and three different gelling b-sheet-forming with various surface chemistries were studied. The mechanical properties of hydro-gels were examined with the inclusion of GDs in a peptide nanofiber matrix for investigating the molecular interactions between the filler and the matrix (Wychowaniec et al., 2018). The effects of coupled hydrophobic interactions and electrostatic and the consequent outcomes of these interactions on the module were evaluated by changing the surface chemistry of the GDs and the peptides physicochemical properties. If both hydrophobic interactions and long-range electrostatic interactions are favorable, G0 increases, and the relative strength of each contribution determines the outcome of G0 when the interactions compete (Wychowaniec et al., 2018).

However, because of geometry, the capability to modulate the drug release from and into micro-domains is limited. Diffusion is restricted with increased particle diameter because spheres have a decreased surface area to volume ratio (Bjørge et al., 2018). Particles with diverse geometries can be created to compensate for this limitation, allowing for maximum surface area and a correspondingly increased range of possible drug release rates. One of such examples is the possibility to improve the release rate of encapsulated Bovine serum albumin (BSA) by using spheroid particles created by squeezing chitosan droplets between two super-amphiphobic surfaces, accompanied by UV-crosslinking. The encapsulation of BSA was accomplished in the particles and a more rapid release from spheroids than from spheres was observed (Bjørge et al., 2018).

The advantages of anisotropic microparticles such as ellipsoids, rods, spheroids, and discs have also shown to apply to hemodynamic environments, with improved marginalization, wall contact, and adhesion suggested by both experimental studies and theoretical modeling (Sen Gupta, 2016). The inclusion of spheroid particles has shown to enhance both the cell viability and diffusion (Bjørge et al., 2018). Increased cell viability and diffusion and the possible hemodynamic transport advantages from anisotropic particles indicate effectiveness for the use of complex anisotropic particles in hybrid hydrogels. Moreover, the non-circular micro-patterned regions in hybrid hydrogels have also revealed an increased absorption of toxin, thus resulting in the detoxification capability of these products (Chen et al., 2017). Hemolytic studies have revealed that star-shaped canals result in a higher detoxification efficiency in comparison to circular canals. This can be attributed to multiple surface plane, increased surface perimeter, and planar area (Chen et al., 2017).

## Recent Trends in the Incorporation of Nano/Microstructures in Hybrid Hydrogels

Several methods have been used to generate hydrogels that involve micronized domains such as emulsion stabilization, photopatterning, and liquid-liquid phase separation, rather than the manufacturing and eventual microparticles incorporation in hydrogels (Garcia and Kiick, 2019). Although all these techniques can create desirable microstructures with different degrees of control, the phase separation has specific advantages due to the one-step manufacture, specificity of the actions, and tunability (Lau et al., 2018). These methods are widely employed to create microstructured resilin-like polypeptide (RLP)-PEG hydrogels. The separation of liquid-liquid phase of RLP and PEG functionalized with acrylate combined by UV-crosslinking is employed for generating RLP-rich hydrogel microdomains distributed in a PEG hydrogel matrix (Lau et al., 2018). High cell viability and direct cell localization around RLP-rich domains are enabled by RLP-PEG hydrogels. By choosing the time point at which the phase-separating solutions are crosslinked after mixing, the domain size can be easily tuned, and the selection of LLPS conditions can provide bicontinuous networks when crosslinks occur during the initial phases of spinodal decomposition (Lau et al., 2018). Although this approach is easily used *in situ* and provides tunability in terms of domain size, there is a restricted range of structures.

Methods like 3D printing and photopatterning are widely used to create hydrogels with detailed and precise microstructures. Techniques like these, however, are resolution-limited and labor-intensive, which limits the versatility of manufacturing and production quality (Nawroth et al., 2018). Techniques, such as such as laser-mediated UV photo-patterning, are less time-consuming and highly precise, and currently being investigated to overcome these limitations (Duan et al., 2019). As an example, photopatterning of riboflavin-50 phosphate gelatin hydrogels as a non-toxic UV photosensitizer has allowed faster development of micropatterned substrates with less variability and spatial resolution compared to conventional photolithographic and micromolding techniques (Nawroth et al., 2018). In combination with crosslinking and casting of polymeric materials, these techniques could be used to produce hybrid hydrogels with domains having a fine tunability. Micro-patterned gels could also be assembled for generating multilayered heterogeneous materials with embedded high-resolution microchannels that enable perfusion (Attalla et al., 2018). The outcome is a hollow microchannel hydrogel (150 mm-1 mm) that enables faster cell viability when evaluated over a 7-day duration by using silicon carbide as an adhesive to promote strong binding between micro-patterned collagen and alginate hybrid hydrogel films (Attalla et al., 2018).

## Applications of Hybrid Hydrogels

### Hybrid Hydrogels in Anticancer Treatment

Cancer stands on top of the deadliest diseases worldwide. About 9.6 million people died of this disease around the world according to 2018 report (Bray, 2018). Surgery, radiotherapy, chemotherapy, and immunotherapy are the cancer treatment strategies applied clinically now a day. Though, chemotherapy seems effective up to some extent, full application of this strategy is hindered by the adverse anticancer drugs' reactions, drug tolerance, low therapeutic index, and effecting other tissues alongside target tissues (Senapati et al., 2018). Hydrogels, being comprised of excessive water components and a cross-linked polymer network endowed with extraordinary biocompatibility, less cytotoxicity, and excellent drug-loading capacities (Li and Mooney, 2016). Thus, they offer numerous desirable characteristics to be used for anticancer drug delivery and have been the research interest for application in cancer drug delivery in recent years (Fang et al., 2018; Wu Q. et al., 2019). The main drawbacks of conventional anticancer delivery methods include frequent administration via intravenous route and systemic distribution. To address these issues, different approaches for fabrication and construction of hybrid hydrogel systems have been practiced in recent years. The subsequent section highlights some of the recent examples used for construction of hybrid hydrogels-based systems for anticancer drug delivery application.

Wu et al. recently have reported manganese dioxide nanosheet hybrid hydrogel and used for an anti-tumor application. Their method of hybrid hydrogel preparation was feasible and convenient, and showed good biocompatibility and is expected to be applied in clinics. They initially prepared a single-sheet manganese dioxide nanosheet layer using simple mechanical stripping mode. Subsequently they crosslinked caffeic acid modified chitosan via an *in-situ* oxidation mode on the surface of the nanosheet and thus obtained the manganese dioxide nanosheet hybrid hydrogel. The resultant hydrogel showed good injectability potential due to the presence of excessive hydrogen bonds and π- π interactions in the hydrogel. In addition, the hydrogel showed good tissue adhesion because of the presence of phenolic hydroxyl groups of the caffeic acid in the system. As the tumor microenvironment possess high concentration of hydrogen peroxide (H_2_O_2_), the manganese dioxide nanosheet hybrid hydrogel catalyzed the H_2_O_2_ decomposition and generated oxygen, thus the tumors' cells hypoxic condition was overcome, sensitivity of cancer cells to anticancer drugs was improved, and the melanoma cells growth was effectively inhibited by combining with the emerging photothermal treatment (Wu M. et al., 2019).

**Figure 2:**
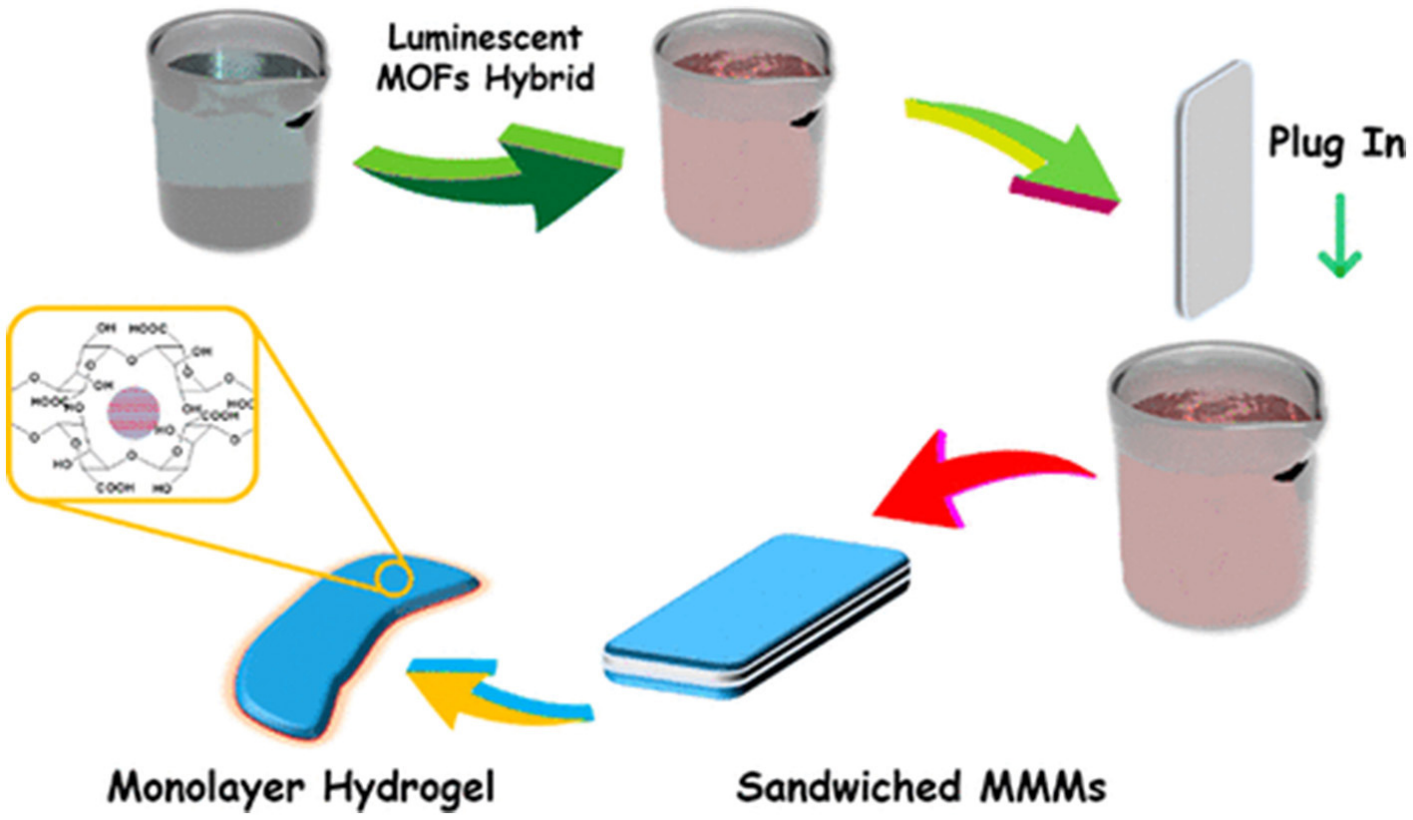
A sandwich of Mixed Matrix Membrane (MMM) on the basis of hybrid hydrogel containing luminescent MOF. Reproduced with permission from Lian et al. (2020). Copyright (2020) American Chemical Society.

Metal organic frame-works (MOFs) have also been encapsulated in hydrogel system to produce hybrid system. In an example, Lian et al. produced such a system and used for the detection of Mitoxantrone Figure 2. Their MOFs of sandwiched mixed matrix membrane (MMM) based framework-hydrogel hybrid system showed excellent mitoxantrone detection performance with sensitivity up to ppb-level (13.4 ppb) and good serum selectivity among other anticancer drugs. Their flexible MMM based system could be employed for point-of-care testing bioactive drugs in biological medium. It was concluded that the MMM can identify mitoxantrone among other different anticancer drugs or their metabolites/ derivatives with nearly similar chemical structures, thus, can be successfully employed for point-of care testing of drugs in biological medium (Lian et al., 2020).

In another example, Javanbakht et al. developed an anticancer drug (5-fluorouracil; 5-FU) encapsulated porous Zn-based MOFs (MOF-5) and embedded in CM-cellulose (CM-cellulose) biopolymer to produce 5-FU@MOF-5 bio nanocomposite hybrid hydrogel bead system coated with CMC. The 5-FU was encapsulated in Zn-based MOFs (MOF-5) via immersion of the MFOs in the drug solution. The pH sensitive biopolymer of CMC was used for the protection and carrying of 5-FU encapsulated MOF-5 nano-hybrid through the digestive tract. The drug release in simulated gastric environment showed that the MOFs containing hybrid hydrogel possessed a sustained release profile and showed noticeable toxicity against HeLa cells in the MTT assay. Their findings showed that the prepared hydrogel beads system could be used as a potential anticancer drug delivery system for 5-FU targeting in colon cancer (Javanbakht et al., 2020).

Schneible et al. constructed a hybrid hydrogel-based system comprised of modified graphene oxide (GO) nanoparticles that were embedded in Max8 peptide hydrogel for controlled kinetics and molar ratios release of anticancer drugs i.e., gemcitabine (GEM) and doxorubicin (DOX). Initially, they modified GO nanoparticles (tGO) for affording high loading sustained release of DOX (19% over 72 h and 31.4% over 4 wk). They also utilized Molecular dynamics (MD) simulations to model the DOX loading mechanism as a function of surface modification. A Max8 based hydrogel was prepared in parallel for the release of GEM with rapid release kinetics and achieved a 10 times higher molar ratio to DOX. These DOX/tGO nanoparticles were then embedded in a hydrogel matrix of GEM/Max8. The developed hybrid system was was also tested against a triple negative breast cancer (TNBC) cell line (MDA-MB-231). The results of their study showed that the composite formulation synergistically enhanced the anticancer effect as compared to the administration of DOXGEM combination in solution form (Schneible et al., 2020).

A Prussian blue (PB) nanoparticles' embedded nanozyme-hydrogel (hPB-gellan) based hybrid injectable hydrogel system was developed by Hao et al. The biomimetic cascade bioreactor was developed for combining antitumor therapy via providing long-term delivery and spatiotemporally-controlled of anticancer agents. The hybrid nanozyme-hydrogel (hPB-gellan) was doped with PB nanoparticles through nanoprecipitation method in the gellan matrix of polysaccharide. The resultant PB nanoparticles were of 10 nm size and displayed dual functions of photothermal agent and as a nanozyme for the decomposition of H_2_O_2_ into oxygen. Due to its self-recovery and shear-thinning properties, the developed hybrid hydrogel was administered locally into tumors and showed resistance against body metabolism and clearance for long-term. *In-vivo*, the antitumor effects of this system showed great elimination of tumors in combined photothermal and starvation therapy (GOD/hPB-gellan + NIR) group with 99.7% tumor suppression rate after 22 days treatment. The enhanced anticancer effect of this system was believed to be due to the NIR-triggered hyperthermia attack and GOD-mediated holding attack starvation from the catalytic bioreactor of the hybrid system. Keeping in view the advantageous properties of biocompatibility, easy manipulation in treatment, and simple synthetic approach, this hybrid hydrogel has great potential for clinics (Hao et al., 2020).

A hybrid Hydrogel system was developed by Capanema et al. composed of CMC-Silver nanoparticles-Doxorubicin for Antibacterial and Anticancer effects. The hybrid hydrogel system was made of silver nanoparticles (AgNPs) embedded in the doxorubicin (DOX)- conjugated CMC polymer crosslinked networks. A green synthetic approach was used for the preparation of this system using a one-pot reduction of Ag^+^ by CMC polymer *in situ* that also worked as capping ligand, followed by DOX conjugation electrostatically in aqueous media. The prepared nano sized conjugates were then crosslinked with citric acid (CA) chemically under mild temperature and pH environments. The prepared hybrid system showed tuned intracellular DOX kinetics *in-vitro*, suggesting synergistic effects for killing melanoma cancer cells with AgNPs. In addition, hybrid system also showed good antimicrobial potentials against different Gram-positive and Gram-negative bacteria. Thus, an innovative hybrid hydrogels platform was produced with anticancer and antibacterial properties and could be successfully applied as a weapon against skin cancer (Capanema et al., 2019).

Paclitaxel (PTX) loaded Quantum dots (QDs) embedded polypeptide hybrid hydrogel system has been developed by Jin et al., The injectable multifunctional hydrogel was prepared for sustained chemo-photothermal cancer therapy. Based on an engineered coiled-coil polypeptide, paclitaxel (PTX) and Ag2S quantum dots (QDs) were designed for chemo-photothermal therapy in a sustained profile. Oil-sol. Initially, hydrogel was prepared with engineered polypeptide (PC10A) and then PTX and Ag2S QDs were loaded into the nanogel via ultrasonic treatment to prepare PC10A/Ag2S QD/PTX nanogel system. Then, PC10A/Ag2S QD/PTX multifunctional hydrogels were constructed by dissolving PC10A/Ag2S QD/PTX nanogels into the previously prepared PC10A hydrogel. The PC10A/Ag2S QD/PTX hydrogel was also suitable for direct injection to the tumor site. *In-vitro* and *in-vivo* toxicity studies showed that prepared hybrid hydrogel system was extremely biocompatible. The combined therapy effectively suppressed the SKOV3 ovarian carcinoma tumor cells growth in comparison with chemotherapy and single near-IR photothermal therapy. Moreover, the system was real-time monitored for *in-vivo* degradation using photoacoustic imaging near-IR fluorescence imaging. Their results showed that the prepared multifunctional injectable PC10A/Ag2S QD/PTX hydrogel had the potential for use as theranostic system for sustained cancer treatments (Jin et al., 2019).

**Figure 3:**
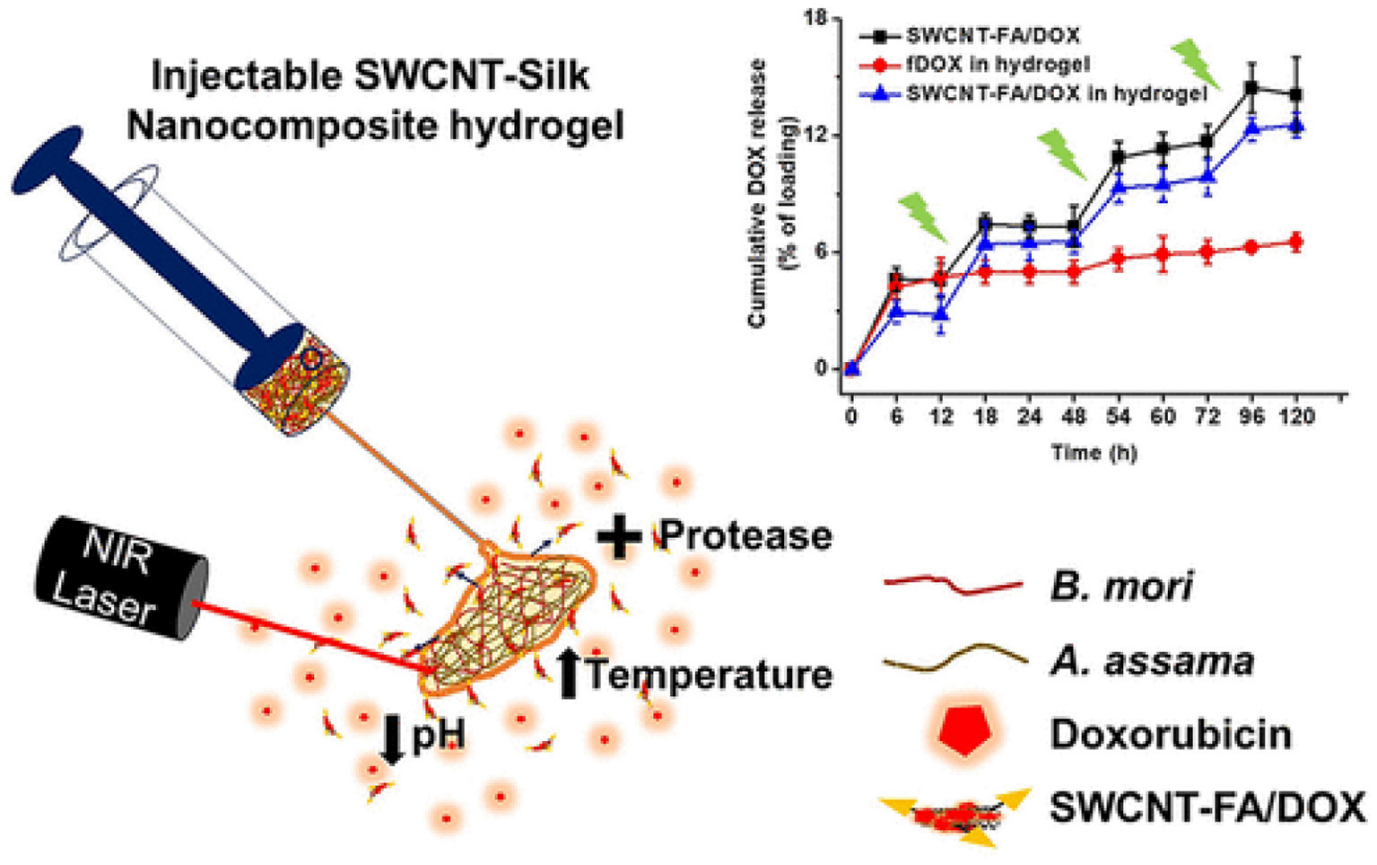
Design and development of injectable multifunctional hydrogel based on carbon nanotube impregnated silk for on-demand targeted anticancer drug delivery. Reproduced with permission from Gangrade and Mandal (2019). Copyright (2019) American Chemical Society.

Gangrade and Mandal prepared a silk based nano hybrid hydrogel system for targeted, localized, and on-demand anticancer drugs' delivery. The hybrid formulation contained (DOX)-loaded folic acid functionalized single-walled carbon nanotubes (SWCNTFA/ DOX) and a blend of two varieties of silk protein. A sustained and slow release of DOX over 14-day study was observed (Figure 3). The DOX release was assessed in different parameters i.e., rate of silk degradation, concentration of the SWCNTFA/ DOX payload, pH of the released medium, and temperature of incubation. DOX release was stimulated with exposure to intermittent near-IR light of the hybrid gel system. Folic acid receptor-positive (FR +ve) cancer cells active targeting of SWCNT-FA/DOX was observed in *in-vitro* studies. Being viscoelastic in nature, the silk hydrogel was easy to inject to the target site. Thus, their prepared silk hybrid hydrogel system could allow its intra-tumoral or near- tumoral implantation, where it will serve as anticancer drug loaded nanoparticles depot (Gangrade and Mandal, 2019).

**Figure 4:**
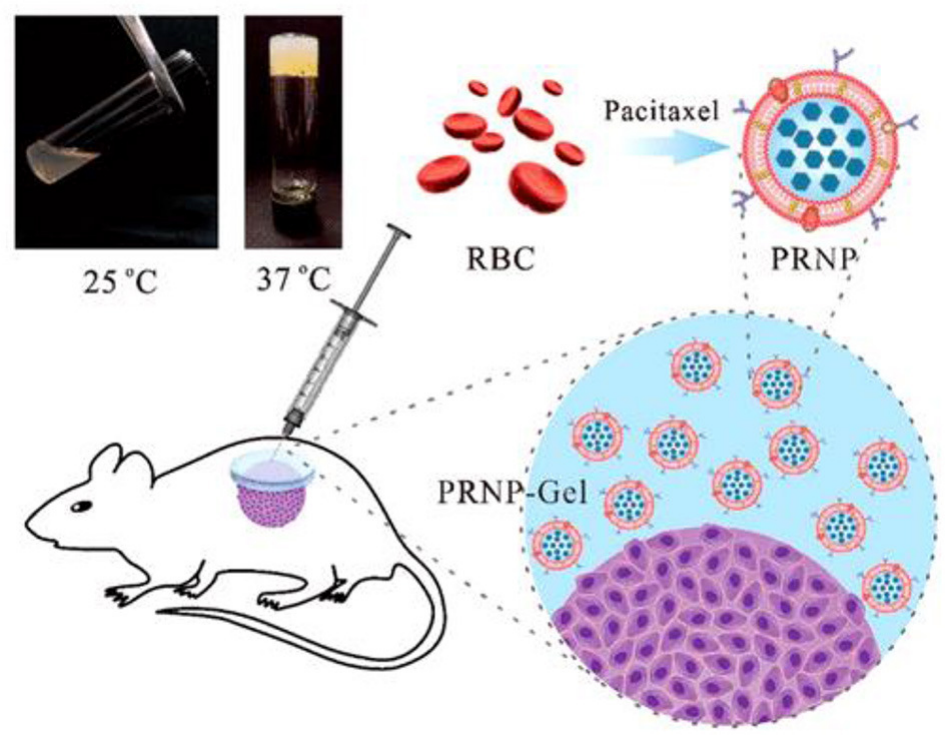
Injectable albumin hydrogel hybridized with paclitaxel-loaded red blood cell membrane nanoparticles for the treatment of gastric cancer with peritoneal metastasis. Reproduced with permission from Qian et al. (2019). Copyright (2019) American Chemical Society.

Similarly, an injectable hydrogel-encapsulating PTX-loaded red blood cell (RBC) membrane nanoparticles (PRNP-gel) was designed by Qian et al. The (PRNP-gel) system was constructed using temperature -induced phase transition of polyethylene-glycol (PEG) modified bovine serum albumin (PEG-BSA). The prepared PRNP were of spherical shape with nearly 133 nm diameter and negative zeta potential. The system drug loading efficiency was found 85% with loading content of 22%. The *in-situ* gelation took 12 min when the gel precursor was injected subcutaneously or incubated at 37°C. A sustained release profile of the drug was shown by the *in-situ*-forming hydrogel and total PTX release after 6 days was around 30%. The PRNP-gel showed excellent *in-vivo* and *in-vitro* biodegradability and cytocompatibility. The nanoparticle-hydrogel hybrid system was used for local chemotherapy as a drug carrier to reduce the systemic toxicity and enhance therapeutic concentrations at tumor site. In peritoneal dissemination and subcutaneous xenograft model, the *in-vivo* anticancer evaluation of this system showed that it possessed excellent tumor growth suppression potential after a single injection (Figure 4). Thus, the prepared injectable hydrogel platform could potentially be used as a promising system for local delivery of anticancer drugs (Qian et al., 2019).

### Hybrid Hydrogels in Tissue Engineering

Hydrogels stands the most attracting candidates among different investigated tissue engineering scaffolds because of their natural extracellular matrix (ECM) structural resemblance, innate biocompatibility, higher water contents, tunable viscoelasticity and high permeability for essential nutrients and oxygen (Lee and Mooney, 2001). The physical properties of hydrogels can easily be manipulated in response to changes in environment and cellular activities (Gil and Hudson, 2004; Kopeček, 2007). Chemically or physically crosslinked hydrogel systems can be constructed in the living cells, thus allow its *in situ* encapsulation for tissue engineering (Sakai et al., 2009; Xing et al., 2011). Different types of hydrogel materials have been used for tissue engineering applications (Raschip et al., 2007, 2013) including reconstituted ECM components or natural proteins and carbohydrates (Beamish et al., 2010; Hejčl et al., 2010; Zustiak et al., 2013), self-assembling peptides (Jiang et al., 2008; Deshmukh et al., 2010), and synthetic hydrogels (Schmedlen et al., 2002; Higuchi et al., 2006; Ossipov et al., 2007). Though, they show unique properties that them as nanostructured scaffolds for tissue engineering. However, several limitations are associated with single-component hydrogels owing to the low versatility of single component used in their formulations. To achieve multi-component hybrid hydrogel-based systems, various approaches have been applied. In an example, Amiryaghoubi et al. developed thermo-sensitive injectable hydrogel system composed of poly (N-isopropylacrylamide) (PNIPAAm) based copolymer/graphene oxide (GO) composite with different chitosan feed ratio through physical and chemical crosslinking. This hybrid hydrogel system was used for the differentiation and proliferation of the human dental pulp stem cells (hDPSCs) to the osteoblasts. The copolymer/GO composite was prepared in the presence of GO using free-radical copolymerization of (Nisopropylacrylamide) (NIPAAm), itaconic acid (IA) and maleic anhydride-modified PEG. It was ultimately used for the construction of hydrogels system. The hydrogel enhanced the deposition of minerals and the activity of alkaline phosphatase (ALP), mainly attributed to the oxygen and amine-containing functional groups of CS and GO. The hydrogel also upregulated the expression of the osteocalcin and Runt-related transcription factor 2 in the hDPSCs cultivated in both osteogenic and normal media and also promoted the absorption of osteogenic inducer. Thus it was proposed to be a constructing scaffold in bone tissue engineering for the transplantation of hDPSCs (Amiryaghoubi et al., 2020).

Recently, Lee et al. synthesized a hyaluronate-alginate hybrid (HAH) hydrogel by introduction of alginate to hyaluronate backbone with different molecular weights (700–2,500 kDa) and HAH hydrogels were designed in the presence of calcium ions. As the molecular weight of the hyaluronate were increased in the HAH polymer, an increase in the storage shear moduli of the hydrogels was observed. Further modification of the HAH hydrogels with histidine-alanine-valine (HAV) and arginine-glycine- aspartic acid (RGD) peptides were induced for enhancing cell-cell interactions and cell-matrix respectively. With an increase in storage shear moduli of the gel, the differentiation (chondrogenic) of ATDC5 cells encapsulated in the HAH hydrogels were increased *in-vitro* and *in-vivo*. Such approach of viscoelastic properties regulation of hydrogels via polymers with varying molecular weights at same crosslinking degree could prove beneficial in different cartilage regeneration and other tissue engineering applications (Lee et al., 2020).

Modifying the ECM based hydrogels properties by conjugation with different synthetic polymers is an emerging strategy for the design of hybrid hydrogels for different tissue engineering applications. In a study, Raj et al. conjugated poly(ethylene glycol) diacrylate (PEGDA) a synthetic polymer with porcine cholecyst derived ECM (C-ECM) (1% wt/vol) at various concentrations (0.2 - 2% wt/vol) and prepared a biosynthetic hydrogel with enhanced physico-mechanical properties for application in skeletal muscle tissue engineering. N-hydroxy succinimide was used for C-ECM functionalization with acrylate groups and it was then conjugated with PEGDA in presence of ammonium persulfate and ascorbic acid through free radical polymerization process. The hydrogel formulation containing PEGDA (0.2 and 0.5% wt/vol) were found suitable for proliferation and growth of skeletal myoblasts and were non-toxic. Their study showed a process for modulation of ECM hydrogels properties via conjugation with bioinert polymers for application in skeletal muscle tissue engineering (Raj et al., 2020).

**Figure 5:**
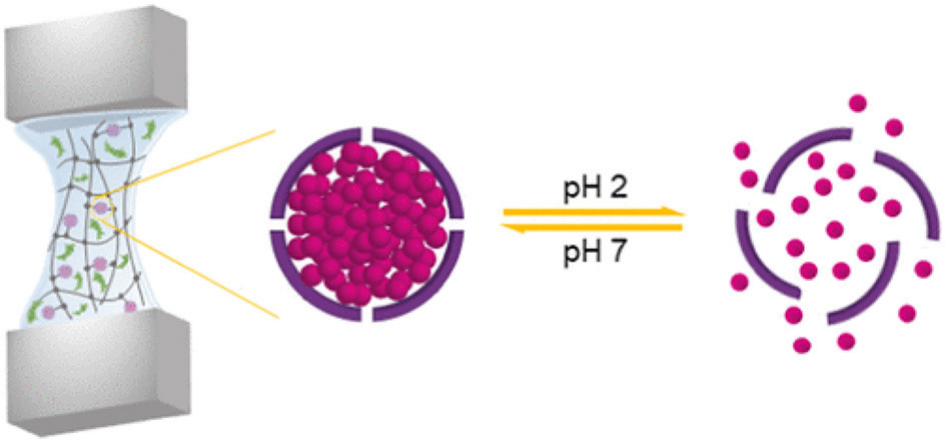
Ferritin nanocage conjugated hybrid hydrogel for tissue engineering and drug delivery applications. reproduced with permission from Samanipour et al. (2019). Copyright (2019) American Chemical Society.

Samanipour et al. developed both covalently and physically conjugated nanocage-laden hydrogels between the gelatin methacryloyl (GelMA) hydrogel matrix and surface of the nanocage. They used Ferritin and its empty-core equivalent apoferritin as nanocages that easily incorporated into a GelMA hydrogel via physical bonding (Figure 5). Apoferritin and Ferritin were modified chemically to present the methacryloyl groups for the fabrication of covalently conjugated nanocage-laden GelMA hydrogels. The covalently conjugated FerMA- and ApoMA-GelMA hydrogels showed better ability for tunable mechanical properties as compared to those systems synthesized via direct ferritin and apoferritin dispersion in GelMA hydrogels with physical bonding. Moreover, a cumulative release test of small molecules on fluorescein isothiocyanate (FITC) encapsulated apoferritin and ApoMA incorporated GelMA hydrogels by pH stimulus was performed. The nanocage incorporated hydrogels showed excellent potential for tissue engineering and drug delivery (Samanipour et al., 2019).

**Figure 6:**
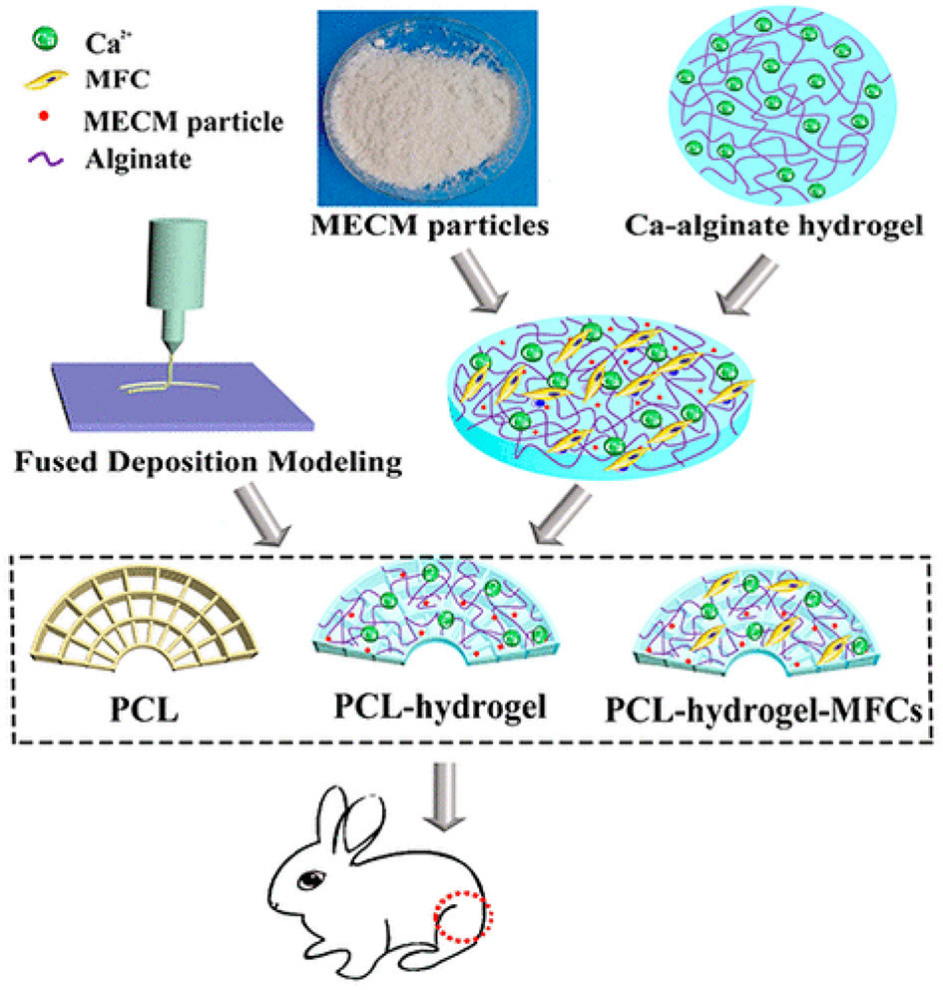
PCL-MECM-Based hydrogel hybrid scaffolds and meniscal fibrochondrocytes promote whole meniscus regeneration in a rabbit meniscectomy model. Reproduced with permission from Chen et al. (2019). Copyright (2019) American Chemical Society.

Chen et al. prepared poly(e-caprolactone) (PCL)- meniscus extracellular matrix (MECM) PCL-MECM-Based Hydrogel Hybrid for Meniscal Fibrochondrocytes loading and used for whole Meniscus regeneration in a Rabbit Meniscectomy Model. They first investigated five concentration (0, 0.5, 1, 2, and 4%) of MECM-based hydrogel for the matrix-forming phenotype of meniscal fibrochondrocytes (MFCs) and promoting cell proliferation and found that 2% strongly enhanced chondrogenic marker mRNA expression and cell proliferation. In addition, the 2% system showed highest glycosaminoglycan (GAG) and collagen production on 14th day. They, finally constructed a hybrid scaffold via 3D printing a wedge-shaped PCL scaffold backbone, followed by injection with the optimized MECM-based hydrogel (2%) that served as cell delivery system. The hybrid PCL-hydrogel scaffold yielded near to native meniscus biomechanical properties. The PCL-hydrogel, PCL scaffold, and MFCs-loaded hybrid scaffold (PCL-hydrogel-MFCs) were finally implanted in the knee joints of rabbits that underwent total medial meniscectomy (Figure 6). After six months, the PCL-hydrogel-MFCs group animals showed extremely better gross appearance and cartilage protection than the other two groups. Thus, the prepared MFCs seeded PCLMECM-based hydrogel hybrid could successfully be used for whole meniscus regeneration, and the cell loaded PCL-MECM-based hydrogel hybrid could be used as a promising technique for regeneration of meniscus in future (Chen et al., 2019).

**Figure 7:**
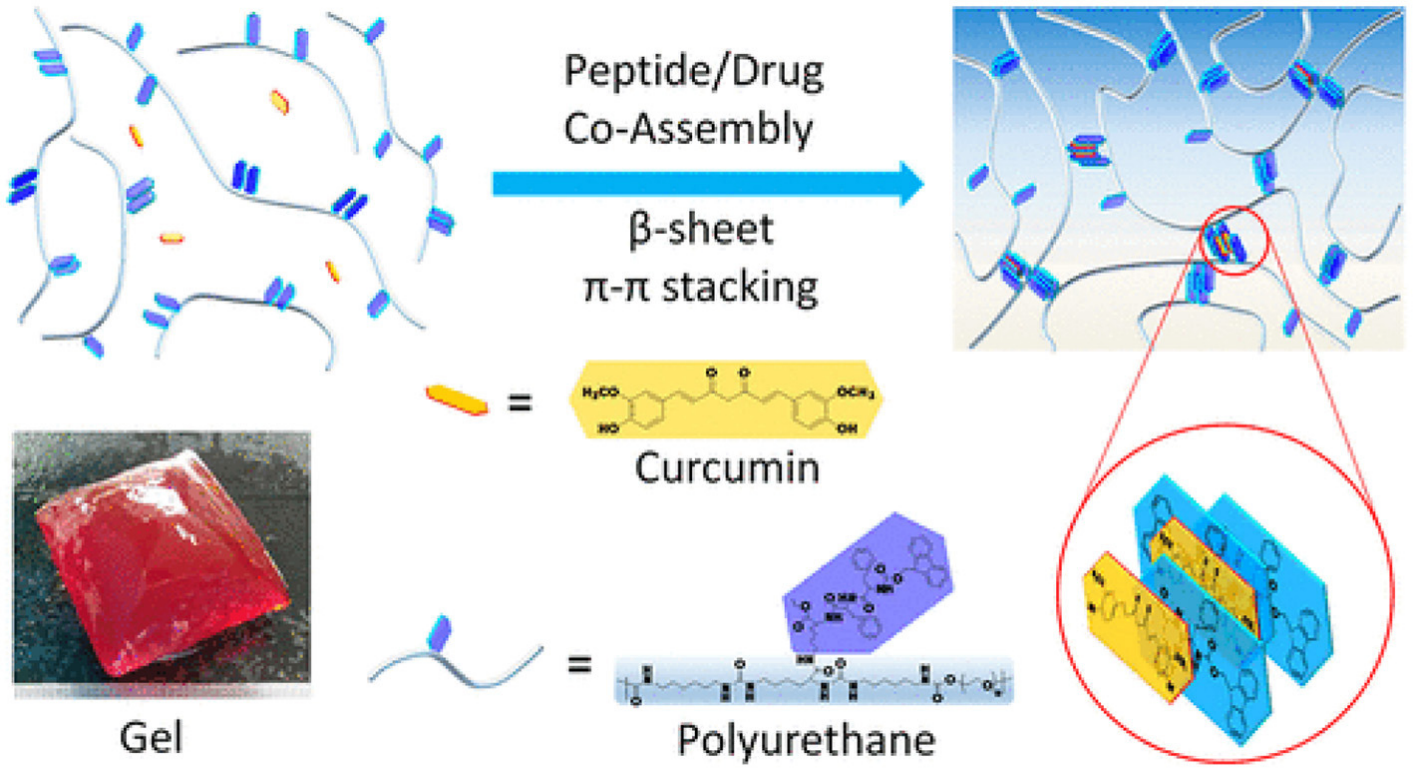
Peptide-/Drug-Directed self-assembly of hybrid polyurethane hydrogels for wound healing. Reproduced with permission from (Zhang et al., 2019). Copyright (2019) American Chemical Society.

Zhang et. al has suggested and designed recently a potent hybrid hydrogel method i.e., “peptide-/drug-directed self-assembly” as shown in Figure 7. The hybrid hydrogel system was synthesized using PEG-based Fmoc-FF peptide hybrid polyurethane, in which curcumin was encapsulated via self-assembly with Fmoc-FF peptide through π-π stacking. Curcumin loading efficiency was improved to 3.3 wt% with sustained release profile from the matrix. Moreover, the mechanical properties of the hydrogel were improved with curcumin loading and became nearly similar to that of the natural soft tissues. In addition, the hybrid hydrogel system was injectable and possessed self-healing potential due to reversible and noncovalent Fmoc-FF peptide/curcumin co-assembly. The hydrogels improved cutaneous wound healing in full-thickness skin defected model *in-vivo*. This new peptide-/drug-directed self-assembly of hybrid hydrogel could potentially be used as a promising system for biomedical application and in tissue engineering (Zhang et al., 2019).

The application of hybrid hydrogel systems with biomedical applications is summarized in Table 1 whereas its status in preclinical and clinical phase in Table 2 below.

**Table 1:** Overview of hybrid hydrogel systems with biomedical applications.

**Hybrid hydrogel system composition**	**Preparation method**	**Application**	**References**
PVA/alginate (Alg)	Physical crosslinking of PVA, followed by chemical crosslinking with alginate	Scaffolds for cartilage tissue engineering	Cho et al., 2005
PVA/SA hydrogel, containing nitrofurazone	FT method	Wound dressing	Kim et al., 2008a
Biodegradable PVA/SA clindamycin-Loaded hydrogel film	Physical crosslinking conducted by the FT method	Wound dressing	Kim et al., 2008b
PVA/Alg (1/1 weight ratio) nanofiber hydrogels	*In situ* crosslinking using citric acid (5 wt%) + curing at 140 C, for 2 h + conditioning at room temperature	Tissue engineering	Stone et al., 2013
PVA/calcium alginate nanofiber web	Electrospinning technique	Wound healing	Tarun and Gobi, 2012
PVA/Alg reinforced with cellulose nanocrystals (CNCs)	Acidic hydrolysis	Scaffolds with good proliferation for fibroblast cells	Kumar et al., 2014, 2017
PVA/HA/CTS hydrogels	Gamma irradiation (5–25 kGy)	Potential application in skin tissue engineering	Zhao et al., 2014
PVA/CS hydrogel loaded with vitamin B12	Physical blending between different portions of PVA and water-soluble CS, followed by treatment with formaldehyde to convert –NH2 group of CS into –N = C group in PVA/CS membranes	Potential biomedical applications	Yang et al., 2004
Minocycline loaded PVA/CS hydrogel films	FT method	Wound dressing	Sung et al., 2010
Gelatin/CS/PVA hydrogels	FT process	Potential for tissue engineering applications	Rodríguez-Rodríguez et al., 2019
CS-PEG co-polymer (CS-g-PEG)	Chemically grafting of monohydroxy PEG onto the CS backbone, using Schiff base and sodium cyanoborohydride chemistry	Potential carrier matrices for a wide range of biomedical and pharmaceutical applications	Bhattarai et al., 2005
Injectable composite scaffold obtained from collagen-coated polylactide micro carriers/CS hydrogel	Physical crosslinking	Tissue engineering applications, particularly in orthopedics	Hong Y. et al., 2008
Carboxyethyl chitosan (CE)/PVA nanofiber mats	Electrospinning of aqueous CE-chitosan/PVA solution	Skin regeneration and healing	Xiao and Zhou, 2003
pH and temperature dual-sensitive hydrogel between glycol chitosan and benzaldehyde modified Pluronic	Schiff base reaction	Drug delivery System	Ding et al., 2010
Methacrylate derivative of CS/poly(ethylene oxide diacrylate) (PEODA)	Photo-crosslinking (intensity of UV light of about 10 mW/cm^2^, at a wavelength of 365 nm)	Cartilage tissue engineering	Li et al., 2004
Injectable hydrogels of thiolated HA and 4-arm PEG-vinyl sulfone	Michael-type addition reaction	Cartilage tissue engineering	Jin et al., 2010
Hybrid (chitosan-g-glycidyl methacrylate) (CS–g–GMA)/xanthan hydrogel	Dissolved CS-g-GMA was mixed with the xanthan solution, under nitrogen gas flow, while keeping the temperature at 50 ± 1C under constant magnetic agitation	Potential for use in biomedical engineering applications	Elizalde-Peña et al., 2013

**Table 2:** Hybrid hydrogels in preclinical and clinical phase.

**Phase**	**Hybrid hydrogel system**	**Application**	**References**
Preclinical	Cholesterol-bearing pullulan (CHP)-W9-peptide	Bone loss disorder	Alles et al., 2009
	Acryloyl group-modified cholesterol-bearing pullulan and pentaerythritol tetra (mercaptoethyl) polyoxyethylene	Tissue engineering	Hashimoto et al., 2015
	Pullulan-*g*-poly(l-lactide) copolymers	Anticancer drug delivery carrier	Seo et al., 2012
	Acrylate group-modified cholesterol-bearing pullulan nanogel (CHPANG) with thiol group-modified poly (ethylene glycol)	Protein delivery	Hasegawa et al., 2009
Clinical	BioAquacare^TM^–a novel soft hydrogel based on the poly(ethylene glycol)- soyprotein conjugates	Wound dressing material assessed in partial- and full-thickness wounds in pigs	Shingel et al., 2006
	CHP	Vaccines	Kawabata et al., 2007; Uenaka et al., 2007; Kageyama et al., 2008

## Conclusions and Future Perspectives

Hydrogels containing nano/microstructures allow the design and development of hybrid hydrogels having multiple functionalities for diverse biomedical applications. The inclusion of particles and domains lead to stimuli-responsive material behavior, targeted drug therapy, tuned cellular response, and improved physical and mechanical properties. Hybrid hydrogels are currently used extensively for targeted cancer chemotherapy as well as tissue engineering applications. However, hybrid hydrogels based pharmaceutical preparations have yet entered the clinical phase. As the research regarding hybrid hydrogels is still at infancy, the development of new systems is going to be inspired by biological motifs and structures that closely resemble the environment, for which they are intended. New research for the facile synthesis of hybrid hydrogels via one-step easy methods will be influential to the field as new biomaterials with tunable properties can be prepared for the targeted and desired applications. As the research and innovations continue for preparation of such hybrid hydrogels, advances in biomedical, chemical, and materials engineering will follow.

## Author Contributions

M-HC, X-YC, and L-QF wrote and collected the data. L-QF and W-LD arranged the data and designed the Manuscript. XY, X-ZM, and P-YH revised the manuscript, design, and supervised the whole study. All authors contributed to the article and approved the submitted version.

## Conflict of Interest

The authors declare that the research was conducted in the absence of any commercial or financial relationships that could be construed as a potential conflict of interest.
